# Clinical Characteristics and Treatment Outcomes of Pathologically Confirmed *Aspergillus* Nodules

**DOI:** 10.3390/jcm9072185

**Published:** 2020-07-10

**Authors:** Noeul Kang, Jiyeon Park, Byung Woo Jhun

**Affiliations:** 1 Division of Pulmonary and Critical Care Medicine, Department of Medicine, Samsung Medical Center, Sungkyunkwan University School of Medicine, Seoul 06351, Korea; varsagod0314@gmail.com; 2 Department of Medicine, Samsung Medical Center, Sungkyunkwan University School of Medicine, Seoul 06351, Korea; science0528@gmail.com

**Keywords:** chronic pulmonary aspergillosis, nodule, *Aspergillus* nodule, treatment

## Abstract

*Aspergillus* nodules represent a subtype of chronic pulmonary aspergillosis, but details on their characteristics and outcomes are limited. We evaluated 80 patients with pathologically confirmed *Aspergillus* nodules between January 2009 and December 2016. The median age of the patients was 59 years, and 46 (58%) were women. Seventy-three (91%) patients were surgically diagnosed with *Aspergillus* nodules and the remaining seven (9%) patients were diagnosed by percutaneous transthoracic needle biopsy. The median long-axis diameter of nodules was 22 mm, and nodules had an internal cavity in 49 (61%) patients. Spiculation and calcification were observed in 20% and 39% of patients, respectively. Ninety percent (18/20) of nodules showed uptake on positron emission tomography. Serum *Aspergillus* precipitin IgG antibody was positive in 42% (10/24) of tested patients. Seventy-three (91%) patients underwent surgery without (*n* = 58) or with (*n* = 15) adjuvant antifungal therapy, and the remaining seven (9%) patients received antifungal therapy alone (*n* = 5) or no treatment (*n* = 2). Three patients experienced postoperative pulmonary complications: pneumothorax, hemoptysis, and acute lung injury (*n* = 1 each). There was no recurrence during the median follow-up period of 36.8 months. In conclusion, surgery could be a treatment strategy worth considering for most *Aspergillus* nodules. However, given that our study population was heterogeneous, further well-designed studies are need.

## 1. Introduction

Chronic pulmonary aspergillosis (CPA) is a slowly progressing pulmonary infection caused by *Aspergillus* species, typically *Aspergillus* fumigatus [1,2]. In general, CPA occurs in middle-aged and elderly immunocompetent individuals with chronic pulmonary diseases (e.g., mycobacterial infection, obstructive lung disease, sarcoidosis, or previous history of thoracic surgery) and there is some in-vitro evidence that patients with CPA may have subtle immune defects that confer predisposition to disease [3,4,5]. CPA shows poor prognosis and, as it is associated with multiple respiratory comorbidities, such as tuberculosis, this became a substantial burden in the developing world [6,7]. CPA typically comprises cavity formation with para-cavitary infiltrates but may appear in various other forms. In recent European guidelines, CPA was divided into several phenotypes: simple aspergilloma/nodules, chronic cavitary or fibrosing pulmonary aspergillosis, and a subacute invasive form [1].

*Aspergillus* nodules represent an uncommon subtype of CPA with single or multiple nodular lesions, with or without cavitation, most of which are smaller than 3 cm [1]. However, the clinical and radiological manifestations of *Aspergillus* nodules are nonspecific, and this form of *Aspergillus* infection is challenging to differentiate from other pulmonary diseases in nodular form, especially lung cancer [8,9]. Indeed, most *Aspergillus* nodules are mistaken for malignancy and are diagnosed based on histological findings after surgical resection [10,11,12]. Moreover, there is limited evidence to support the use of serum *Aspergillus* precipitin IgG antibody test for the diagnosis of *Aspergillus* nodules, although it is recommended as a keystone for the diagnosis of CPA [1,13,14,15]. In addition, there is a lack of data regarding the prognosis of *Aspergillus* nodules.

Therefore, it is important to understand the detailed features of *Aspergillus* nodules in clinical practice, especially to avoid unnecessary interventions. However, previous publications have been mostly limited to case reports; only minimal data are available regarding the outcomes of *Aspergillus* nodules under the current definition. Hence, the present study was performed to determine the clinical characteristics and treatment outcomes of pathologically confirmed *Aspergillus* nodules.

## 2. Materials and Methods

### 2.1. Study Population

We retrospectively screened consecutive adult patients (older than 20 years of age) with *Aspergillus* nodules, which were pathologically confirmed by surgical resection or percutaneous transthoracic needle biopsy (PCNB) between January 2009 and December 2016 at Samsung Medical Center (a 1979-bed referral hospital in Seoul, Republic of Korea). *Aspergillus* nodules were defined as discrete, small, round, focal opacities on chest computed tomography (CT), which were further divided into two groups according to the absence or presence of internal cavitation (i.e., non-cavitary nodules and cavitary nodules, respectively) [16]. Patients with other subtypes of CPA (e.g., simple aspergilloma, chronic cavitary or fibrosing pulmonary aspergillosis, and subacute invasive disease) were excluded. Finally, 80 patients with *Aspergillus* nodules were included in the analysis. After surgical resection or PCNB was done, patients were followed-up with either chest X-rays or CT scans at least once in the out-patient clinic. The antifungal agents were used at the discretion of the attending physician. During the follow-ups, a relapse was defined as increased in size or the recurrence of the nodule. The Institutional Review Board of Samsung Medical Center approved the review and publication of information obtained from the patients’ records (approval no. 2019-09-036-001). The requirement for informed consent was waived because of the retrospective nature of the study.

### 2.2. Clinical and Laboratory Evaluation

Clinical and demographic characteristics of the patients (e.g., age, sex, smoking habit, body mass index, and comorbidities) were collected. Data regarding inflammatory markers at the time of diagnosis of *Aspergillus* nodules (e.g., white blood cell WBC] count, erythrocyte sedimentation rate [ESR], and C-reactive protein [CRP]) were analyzed. Fungal culture results of sputum, bronchoalveolar lavage fluid, or tissue at diagnosis were also evaluated. Tests for serum *Aspergillus* precipitin IgG antibody and/or serum *Aspergillus* galactomannan antigen were performed at the discretion of the attending physician at the time of diagnosis. The presence of serum *Aspergillus* precipitin IgG antibody was evaluated using an *A. fumigatus* IgG ELISA kit (IBL International, Hamburg, Germany). The results were reported as positive (>12 U/mL), negative (<8 U/mL), or equivocal (8–12 U/mL). Serum *Aspergillus* galactomannan antigen was assessed using a Platelia *Aspergillus* antigen kit (Bio-Rad, Hercules, CA, USA) and index values were reported as positive (>0.55), negative (<0.45), or equivocal (0.45–0.55) [17].

### 2.3. Radiological and Histological Evaluation

All patients underwent chest CT within one month before the diagnosis of *Aspergillus* nodules. Two of the authors (N.K. and J.P.) reviewed the CT results. The maximal long-axis diameter of the nodule was measured in the axial view in the lung setting. The following CT findings were analyzed: location, margin of nodules, distribution, the presence of calcification and satellite lesions. A central lesion was defined as a lesion which was located inside the half of the longest diameter of thorax in the coronal view of chest CT scan. Likewise, peripheral lesion was defined when it was located outside of the half of the longest diameter. All disagreements were resolved by discussion and subsequent consensus. Results of ^18^F-fluorodeoxyglucose positron emission tomography (FDG-PET) for *Aspergillus* nodules were available for 20 patients, and a maximum standardized uptake value (SUV_max_) > 2.5 was considered significant. Expert lung pathologists reviewed all pathology specimens. Special staining with methenamine silver stain and Periodic Acid-Schiff stain was performed in addition to routine H&E staining. Special stains were used to identify specific microorganisms.

### 2.4. Statistical Analysis

Data are presented as medians (interquartile ranges) for continuous variables and numbers (percentages) for categorical variables. For the comparison of characteristics between patients with non-cavitary nodules and patients with cavitary nodules, Student’s *t*-test was used for continuous variables and the Pearson Chi-squared test was used for categorical variables. All tests were two-sided, and a *p* < 0.05 was considered statistically significant. All analyses were performed using Stata software (version 14.0; Stata Corporation, College Station, TX, USA).

## 3. Results

### 3.1. Baseline Characteristics

The characteristics of the 80 patients with pathologically confirmed *Aspergillus* nodules are presented in Table 1. Seventy-three patients (91%s) were surgically diagnosed with *Aspergillus* nodules, and the remaining seven patients (9%) were confirmed by PCNB without surgery. Thirty-one patients (39%) had non-cavitary nodules, while 49 patients (61%) had cavitary nodules. The median age of the study population was 59 years (interquartile range, 48–65 years), and 46 (58%) were women. More than 70% of patients had never smoked. The most common underlying disease was previous pulmonary tuberculosis (49%), followed by bronchiectasis (14%), diabetes mellitus (13%), and malignancy (11%). No patients were treated with steroids, including inhaled corticosteroids. Nearly three-quarters (77%) of the patients had respiratory symptoms, most commonly, hemoptysis (*n* = 54, 68%). Notably, 23% of the patients had no symptoms at diagnosis.

The median WBC count and ESR were 12,400/μL and 23 mm/h, respectively. Of patients for whom serum *Aspergillus* galactomannan antigen (*n* = 26) and serum *Aspergillus* precipitin IgG antibody (*n* = 24) data were available, positive rates were 31% (8/26) and 42% (10/24), respectively. Fungus culture was positive in 26% of 46 patients who underwent fungus culture using respiratory specimens, with positive results in 50% (6/12) of sputum, 17% (2/12) of bronchoalveolar lavage fluid, and 50% (6/12) of lung tissue specimens. Overall, there were no significant differences in clinical or laboratory findings between patients with non-cavitary nodules and patients with cavitary nodules, except for WBC counts (8700/μL vs. 12,900/μL, respectively, *p* = 0.029).

### 3.2. Radiological and Histological Characteristics of Aspergillus Nodules

**Figure 1:**
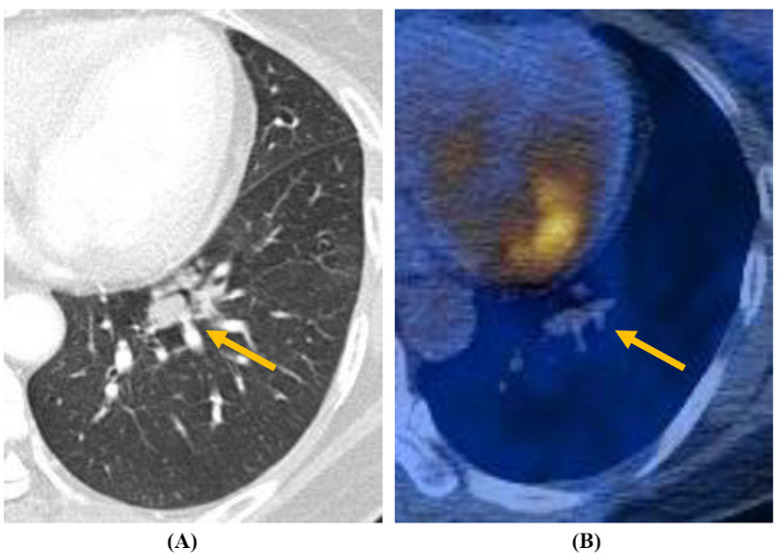
Cavitary *Aspergillus* nodule (arrowhead) from a 62-year-old woman. (**A**) Chest CT revealed a 23 mm round nodule in the left lower lobe. (**B**) PET/CT uptake showed a SUV_max_ value of 2.0.

**Figure 2:**
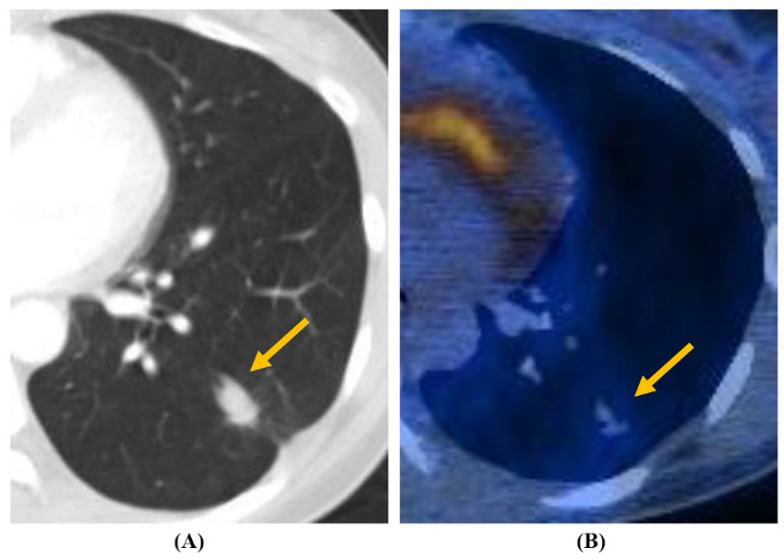
Non-cavitary *Aspergillus* nodule (arrowhead) from a 45-year-old woman. (**A**) Chest CT showed a 16 mm smooth oval nodule in the peripheral portion of the left lower lobe. (**B**) PET/CT uptake showed a SUV_max_ value of 1.2.

The radiological characteristics of *Aspergillus* nodules are summarized in Table 2, and the typical findings of *Aspergillus* nodules with or without an internal cavity are shown in Figure 1 and Figure 2, respectively. Most (98%) of the patients had a solitary nodule, whereas 2% had multiple nodules. The median long-axis diameter of all nodules on chest CT was 22 mm (interquartile range, 16–31 mm). The nodules were located in the right, left, upper, and lower lobes at similar rates. Approximately half of the nodules (51%) were distributed in peripheral areas. Spiculation (or lobulation) and calcification were observed in 20% and 39% of the nodules, respectively. Only 48% of all nodules were visible on chest radiographs. Of the 20 nodules for which FDG-PET/CT data were available, 90% (18/20) had relatively high uptake (SUV_max_ >2.5). However, there were no significant differences in radiological characteristics between patients with non-cavitary nodules and patients with cavitary nodules.

### 3.3. Treatment Modalities and Outcomes

Treatment modalities and outcomes of patients with *Aspergillus* nodules are shown in Table 3. Of 73 patients who underwent surgical resection for *Aspergillus* nodule(s), most patients (79%, 58/73) received no additional antifungal therapy, and approximately half of these patients (52%, 30/58) had received sublobar resection. Fifteen patients (21%, 15/73) who had surgical resection received subsequent additional antifungal therapy, and 12 of these 15 patients underwent lobectomy or sublobar resection. The nodules were incidentally confirmed in the remaining three of 15 patients. One patient underwent lung transplantation due to advanced interstitial pulmonary fibrosis related to Sjögren’s disease. The other two patients underwent completion pneumonectomy, due to severe hemoptysis after prior lobectomy.

**Figure 3:**
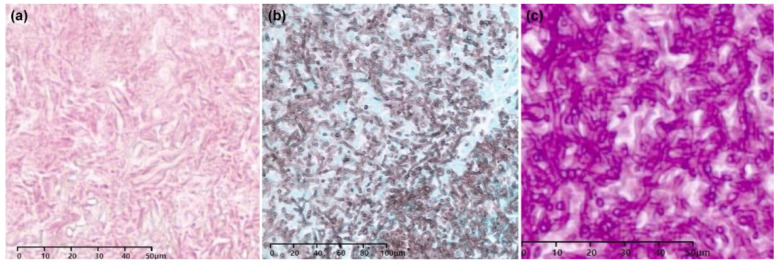
Surgical specimen of a non-cavitary *Aspergillus* nodule featuring dichotomous branching and hyphae with frequent septation. (**a**) Hyphae were observed as translucent material on hematoxylin and eosin staining. (**b**) Grocott’s methenamine silver staining. (**c**) Periodic Acid–Schiff staining.

Five patients (6%, 5/80) received antifungal therapy alone, and two patients (3%, 2/80) received no additional treatment after confirmation of a diagnosis of *Aspergillus* nodule by PCNB, without surgical resection. Of the 73 patients who underwent surgical resection, surgery was performed via thoracotomy in 21 patients and video-assisted thoracoscopic surgery in 52 patients. Representative histological findings of *Aspergillus* nodules are shown in Figure 3.

For the 20 patients who received antifungal therapy, the median duration of antifungal treatment was 5.3 months (interquartile range, 2.3–8.5 months). Most patients (95%, 19/20) received itraconazole, while only one patient (5%, 1/20) received voriconazole. Three patients experienced postoperative pulmonary complications: pneumothorax, hemoptysis, and acute lung injury (*n* = 1 each). There were no significant differences in postoperative complications between patients with non-cavitary nodules and patients with cavitary nodules.

There were no recurrences of *Aspergillus* infection during the median follow-up period of 36.8 months (interquartile range, 16.0–70.5 months). All seven of the patients who did not have resection had follow-up chest CT scans—two patients without treatment showed no change in the nodule after a one-year follow-up and the other five patients who had been treated with antifungals showed a decrease in nodule size in follow-up scans after treatment. Among the 10 patients who had positive results on *Aspergillus* precipitin IgG antibody tests before surgical resection, four patients had serial follow-up tests. Two patients had decreased titers after the surgical removal of their *Aspergillus* nodule(s), but showed elevated titers later on during follow-up, although there was no evidence of *Aspergillus* infection. The other two patients showed elevated antibody titers even after the removal of their *Aspergillus* nodules.

## 4. Discussion

*Aspergillus* nodules represent an uncommon subtype of CPA and are a clinical problem because they often mimic other diseases, such as malignancy or inflammatory nodules [1,9,10]. In this study, we comprehensively reviewed patients with pathologically confirmed *Aspergillus* nodules. Most of our patients were middle-aged with normal body mass index and had symptoms such as hemoptysis (68%). Inflammatory markers showed nonspecific findings; however, the WBC count tended to be slightly higher in patients with cavitary *Aspergillus* nodules. The median diameter of the nodules was <3 cm, and most nodules had smooth margins (80%) without calcification (61%) on CT, although more than half (52%) of the nodules were not visible on radiographs. There were no significant differences in clinical–radiological features or prognosis between patients with cavitary nodules and those with non-cavitary nodules. Attempts have been made to establish detailed features and treatment outcomes of *Aspergillus* nodules, but previous publications have largely consisted of case series or small observational studies [9,10,12]. Although a recent study performed in the U.K. involved 33 immunocompetent patients with *Aspergillus* nodules [8], only limited data are available regarding the characteristics of *Aspergillus* nodules. Therefore, our data provide insight in this context.

A notable finding of the present study was that patients with *Aspergillus* nodules had favorable outcomes, regardless of the presence or absence of internal cavitation. Most patients could be treated completely by surgical resection without perioperative antifungal therapy, and two of our patients received no treatment after *Aspergillus* nodules were diagnosed using a nonsurgical approach. Furthermore, no patients exhibited recurrent pulmonary *Aspergillus* infection during the median follow-up period of 36.8 months. These findings were consistent with the results of previous studies, in terms of favorable outcomes for patients with *Aspergillus* nodules [18]. Because *Aspergillus* nodules in the present study generally manifested as localized, solitary nodules, our data suggest that complete surgical resection of *Aspergillus* nodules may be curative in the absence of other remnant lesions presented radiographically. In addition, although small in number, our data may suggest that conservative management with close observation alone could be a treatment strategy worth considering, especially in patients for whom surgery is a high-risk approach.

With regard to antifungal therapy for *Aspergillus* nodules, limited data are available concerning optimal treatment duration or on the benefits of additional antifungal therapy after surgical resection [2,19]. Current guidelines indicate that when the *Aspergillus* nodule is single and completely resected, antifungal therapy is not required for non-immunocompromised patients [1]. However, a subset of patients with *Aspergillus* nodules may have chronic inflammatory pulmonary or extra-pulmonary disease, which requires treatment with immunomodulatory drugs. As no guidance has been established regarding a beneficial role for antifungal therapy, further studies are needed to determine treatment strategies with regard to the use of antifungal agents for the management of *Aspergillus* nodules. We cautiously suggest that the use of antifungal therapy could be individualized based on preoperative clinical symptoms or postoperative outcome.

In our study, postoperative complications occurred in three of the 73 patients who underwent surgery. One patient exhibited mild pneumothorax after hospital discharge and two patients had severe hemoptysis and acute lung injury. The patient with pneumothorax recovered quickly after the insertion of a chest tube, but the other two patients underwent segmentectomy/lobectomy and received mechanical ventilation in the intensive care unit. Although the postoperative complications for these two patients were eventually fatal, there were no mortalities during the study period and the incidence of therapy-related complications was lower than with other benign lung diseases [20,21,22]. Various complications associated with aspergilloma resection are known to occur, including persistent air leakage, empyema, pneumonia, bronchopleural fistula, respiratory failure, massive hemorrhage, and death [23]; thus, patients should be carefully selected before undergoing surgery. However, there is a lack of data regarding the outcomes of surgical treatment of *Aspergillus* nodules. Therefore, our data may provide more precise information regarding this topic.

In the current guidelines, the detection of serum *Aspergillus* precipitin IgG antibody is a key diagnostic criterion for CPA [1,24]. Unfortunately, it is unclear whether the presence of *Aspergillus* IgG antibody could also be a supportive finding in the diagnosis of *Aspergillus* nodules. In the present study, only a small number of patients had been tested for serum *Aspergillus* precipitin IgG antibody; however, fewer than half (10/24, 42%) of these patients had positive results. These observations suggest that the *Aspergillus* IgG antibody test may be unsuitable for the diagnosis of *Aspergillus* nodules, due to its low sensitivity. Similarly, Muldoon et al. reported that *Aspergillus* precipitin was positive in only four of 32 patients with *Aspergillus* nodules [8]. Thus, further studies are needed to clearly establish the role of the test in the diagnosis of *Aspergillus* nodules.

In our study, *Aspergillus* nodules showed no distinctive features on chest CT that could enable them to be distinguished from malignant or other inflammatory nodules. In addition, 90% (18/20) of the *Aspergillus* nodules for which data were available showed high SUV_max_ uptake on FDG-PET/CT. As malignant nodules or granulomatous nodules, due to tuberculosis or nontuberculous mycobacteria, can show positive signals on PET [25], our data suggested that radiological imaging alone is unlikely to be useful for the differentiation of *Aspergillus* nodules from other etiologies [26]. Thorough investigations, including preoperative histological confirmation, should be considered before undertaking surgical resection to avoid unnecessary intervention or the misdiagnosis of benign conditions [9,26].

This study had several potential limitations. First, it was a single-center, retrospective study. However, this is the largest study in patients with *Aspergillus* nodules reported thus far, despite the low prevalence of CPA. Second, the serum *Aspergillus* precipitin IgG antibody test was performed in less than half of the study population. Although the low sensitivity may have been overestimated, due to the small number of patients, the low sensitivity of the serum *Aspergillus* precipitin IgG antibody test indicates the need for novel biomarkers for the diagnosis of *Aspergillus* nodules. Third, the follow-up period may have been insufficient to evaluate long-term prognosis. Fourth, because approximately half of our patients had an underlying respiratory disease, some of the presented symptoms in our study patients may not be caused by *Aspergillus* nodules, but by underlying diseases. Lastly, as our study population was based on patients who could undergo invasive procedures, there may be a selection bias.

In conclusion, surgery could be a treatment strategy worth considering for most *Aspergillus* nodules. However, given that our study population was heterogeneous, further well-designed studies are need.

## Figures and Tables

**Table 1:** Characteristics of patients with pathologically confirmed *Aspergillus* nodules.

Characteristics	Total (*n* = 80)	Non-Cavitary Nodules (*n* = 31)	Cavitary Nodules (*n* = 49)	*p*-Value
Age, years	59 (48–65)	61 (50–70)	56 (48–62)	0.058
Female	46 (58)	15 (48)	31 (63)	0.190
Never smoker	57 (71)	23 (74)	34 (69)	0.644
Body mass index, kg/m^2^	24.0 (22.1–25.6)	24.0 (22.4–25.9)	23.9 (22.1–25.6)	0.280
Previous pulmonary tuberculosis	39 (49)	12 (39)	27 (55)	0.153
Bronchiectasis	11 (14)	6 (19)	5 (10)	0.322
Nontuberculous mycobacteria	6 (8)	2 (6)	4 (8)	1.000
COPD/asthma	3 (4)	2 (6)	1 (2)	0.556
Interstitial lung disease	3 (4)	1 (3)	2 (4)	1.000
Diabetes mellitus	10 (13)	5 (16)	5 (10)	0.498
Chronic liver disease	4 (5)	1 (3)	3 (6)	1.000
Chronic kidney disease	2 (3)	0	2 (4)	0.519
Malignancy^¶^	9 (11)	6 (19)	3 (6)	0.082
Symptoms at diagnosis ^†^				
Hemoptysis	54 (68)	17 (55)	37 (76)	0.054
Cough	32 (40)	9 (29)	23 (47)	0.111
Sputum	5 (6)	2 (6)	3 (6)	1.000
No symptoms	18 (23)	10 (32)	8 (16)	0.096
Laboratory findings				
WBC/μL	12,400 (7600–14,800)	8700 (6100–14,100)	12,900 (9900–15,000)	0.029
ESR, mm/h (*n* = 42)	23 (14–42)	25 (15–38)	20 (11–44)	0.936
CRP, mg/dl (*n* = 63)	0.11 (0.04–0.41)	0.12 (0.03–0.41)	0.11 (0.05–0.26)	0.054
Positive serum galactomannan (*n* = 26)	8/26 (31)	4/8 (50)	4/18 (22)	0.491
Positive serum *Aspergillus* antibody (*n* = 24)	10/24 (42)	2/8 (25)	8/16 (50)	0.301
Fungus culture (*n* = 46)^§^	12 (26)	2 (4)	10 (22)	0.115
Sputum	6/12 (50)	1/2 (50)	5/10 (50)	0.397
Bronchial washing	2/12 (17)	0	2/10 (20)	0.519
Lung tissue	6/12 (50)	1/2 (50)	5/10 (50)	0.397

Data are presented as number (%) or median (interquartile range). ^¶^ Colorectal cancer (*n* = 4), stomach cancer (*n* = 2), breast cancer (*n* = 1), cholangiocarcinoma (*n* = 1), and lung cancer (*n* = 1). ^†^ 30 patients had more than one symptom at diagnosis. § One patient had positive culture results from both sputum and bronchoalveolar lavage fluid specimens. Another patient had positive culture results from both sputum and lung tissue specimens. COPD, chronic obstructive pulmonary disease; CRP, c-reactive protein; ESR, erythrocyte sedimentation rate; WBC, white blood cells.

**Table 2:** Radiological and histological findings of *Aspergillus* nodules.

Findings	Total (*n* = 80)	Non-Cavitary Nodules (*n* = 31)	Cavitary Nodules (*n* = 49)	*p*-Value
Number of nodules				1.000
1	78 (98)	30 (97)	48 (98)	
≥2	2 (3)	1 (3)	1 (2)	
Size (mm)^¶^	22 (16–31)	20 (15–30)	23 (17–34)	0.631
>3 cm	21 (26)	6 (19)	15 (31)	0.198
≤3 cm	59 (74)	25 (81)	34 (69)	
Location				0.496
Right upper lobe	20 (25)	6 (19)	14 (29)	
Right lower lobe	23 (29)	11 (35)	12 (24)	
Left upper lobe	17 (21)	5 (16)	12 (24)	
Left lower lobe	20 (25)	9 (29)	11 (22)	
Distribution of nodules				0.332
Central	39 (49)	13 (42)	26 (53)	
Peripheral	41 (51)	18 (58)	23 (47)	
Margin of nodules				0.491
Smooth	64 (80)	26 (84)	38 (78)	
Spiculated or lobulated	16 (20)	5 (16)	11 (22)	
Calcification in nodules	31 (39)	9 (29)	22 (45)	0.156
Satellite lesions	21 (26)	8 (26)	13 (27)	0.943
Lymph node enlargement (≥1 cm)	7 (9)	3 (10)	4 (8)	0.815
Visible on chest radiographs	38 (48)	14 (45)	24 (49)	0.739
PET uptake (SUV_max_ ≥ 2.5) (*n* = 20)	18/20 (90)	8 (40)	10 (50)	0.224
Granuloma on histology	20 (25)	5 (16)	15 (31)	0.115

Data are presented as number (%) or median (interquartile range). ^¶^ Long-axis diameter. PET, positron emission tomography; SUV_max_, maximum standardized uptake.

**Table 3:** Treatment modalities and outcomes of patients with *Aspergillus* nodules.

Characteristics	Total *n* = 80)	Non-Cavitary Nodules (*n* = 31)	Cavitary Nodules (*n* = 49)	*p*-Value
Modality for diagnosis				0.012
PCNB	7 (9)	6 (19)	1 (2)	
Surgical resection	73 (91)	25 (81)	48 (98)	
Treatment modality^¶^				0.042
Surgery only	58 (73)	19 (61)	39 (80)	
Lobectomy	28/58 (48)	6/19 (32)	22/39 (56)	
Sublobar resection	30/58 (52)	13/19 (68)	17/39 (44)	
Surgery + antifungal agent	15 (19)	6 (19)	9 (18)	
Pneumonectomy	2/15 (13)	0	2/9 (22)	
Lobectomy	6/15 (40)	2/6 (23)	4/9 (44)	
Sublobar resection	6/15 (40)	4/6 (67)	2/9 (22)	
Lung transplantation	1/15 (7)	0	1/9 (11)	
Antifungal agent only^§^	5 (6)	4 (13)	1 (2)	
No treatment^§^	2 (3)	2 (6)	0	
Antifungal agent (*n* = 20)				1.000
Itraconazole	19/20 (95)	10 (100)	9 (90)	
Voriconazole	1/20 (5)	0 (0)	1 (10)	
Postoperative complications	3/73 (4)	1/25 (4)	2/48 (4)	1.000
Pneumothorax	1/3 (33)	1 (100)	0	
Hemoptysis	1/3 (33)	0	1/2 (50)	
Acute lung injury	1/3 (33)	0	1/2 (50)	
Duration of antifungal agent	5.3 (2.3–8.5)	4.8 (1.9–8.0)	5.3 (2.6–9.0)	0.586
Follow-up, months	36.8 (16.0–70.5)	43.3 (14.0–70.9)	35.6 (20.4–69.9)	0.886

Data are presented as number (%) or median (interquartile range). ^¶^ Surgery was performed via thoracotomy in 21 patients and video-assisted thoracoscopic surgery in 52 patients. One patient was diagnosed with interstitial lung disease related to Sjögren’s syndrome and underwent bilateral lung transplantation due to interstitial lung disease; the presence of *Aspergillus* nodules was determined during lung transplantation. ^§^ Antifungal agent alone (*n* = 5) and no treatment (*n* = 2) groups were diagnosed via PCNB, not surgical resection. PCNB, percutaneous transthoracic cutting needle biopsy.
